# Ultra-processed family foods in Australia: nutrition claims, health claims and marketing techniques

**DOI:** 10.1017/S1368980017001148

**Published:** 2017-07-17

**Authors:** Claire Elizabeth Pulker, Jane Anne Scott, Christina Mary Pollard

**Affiliations:** 1 School of Public Health, Curtin University, GPO Box U1987, Perth, WA 6845, Australia; 2 Department of Health in Western Australia, East Perth, WA, Australia

**Keywords:** Ultra-processed foods, Nutrition labelling, Health claims, Nutrition claims, Marketing, Sugar

## Abstract

**Objective:**

To objectively evaluate voluntary nutrition and health claims and marketing techniques present on packaging of high-market-share ultra-processed foods (UPF) in Australia for their potential impact on public health.

**Design:**

Cross-sectional.

**Setting:**

Packaging information from five high-market-share food manufacturers and one retailer were obtained from supermarket and manufacturers’ websites.

**Subjects:**

Ingredients lists for 215 UPF were examined for presence of added sugar. Packaging information was categorised using a taxonomy of nutrition and health information which included nutrition and health claims and five common food marketing techniques. Compliance of statements and claims with the Australia New Zealand Food Standards Code and with Health Star Ratings (HSR) were assessed for all products.

**Results:**

Almost all UPF (95 %) contained added sugars described in thirty-four different ways; 55 % of UPF displayed a HSR; 56 % had nutrition claims (18 % were compliant with regulations); 25 % had health claims (79 % were compliant); and 97 % employed common food marketing techniques. Packaging of 47 % of UPF was designed to appeal to children. UPF carried a mean of 1·5 health and nutrition claims (range 0–10) and 2·6 marketing techniques (range 0–5), and 45 % had HSR≤3·0/5·0.

**Conclusions:**

Most UPF packaging featured nutrition and health statements or claims despite the high prevalence of added sugars and moderate HSR. The degree of inappropriate or inaccurate statements and claims present is concerning, particularly on packaging designed to appeal to children. Public policies to assist parents to select healthy family foods should address the quality and accuracy of information provided on UPF packaging.

Packaging of foods and non-alcoholic beverages (referred to as ‘food’ hereafter) is an important marketing tool used by manufacturers to communicate product attributes to potential consumers^(^ ^1^ ^)^, with product claims a key feature^(^ ^2^ ^)^. A large proportion of supermarket purchases are made on impulse and packaging has been shown to play a crucial role^(^ ^3^ ^)^. Shoppers typically make these decisions after only a few seconds to consider food labels^(^ ^4^ ^)^. The front of the package plays a vital role in capturing consumers’ attention and influencing food preferences^(^ ^2^ ^,^ ^5^ ^)^. Packaging design can also influence consumer perceptions of health through use of colour and graphical elements such as pictures or symbols^(^ ^6^ ^,^ ^7^ ^)^.

The global food supply has become more concentrated, with major transnational food manufacturers becoming larger and more powerful^(^ ^8^ ^,^ ^9^ ^)^. Researchers have accused the globalised food system, driven by large manufacturers and supermarket chains, of creating processed foods that are identical throughout the world^(^ ^10^ ^)^. They suggest that the extent and purpose of food processing forms the basis of a classification system for use in dietary guidance^(^ ^11^ ^)^. Industrially processed foods that include cosmetic or sensory additives such as colours, flavours, sweeteners, or processing aids, or undergo industrial processes which have no domestic equivalent such as extrusion, also referred to as ultra-processed foods (UPF), have been found to have higher saturated fat, sugar and sodium content compared with less processed foods^(^ ^12^ ^,^ ^13^ ^)^. UPF have also been described as hyper-palatable products that are attractively packaged and aggressively marketed, including making use of health statements and claims^(^ ^12^ ^)^.

In Australia, there is a high level of foreign ownership of food brands by transnational food manufacturers^(^ ^14^ ^)^. UPF are prevalent, with annual retail sales per capita of 200·5 kg in 2013, and Australia ranked sixth out of eight nations for total annual UPF sales^(^ ^15^ ^)^. The majority (83 %) of available packaged foods in New Zealand are UPF, with multiple variations of the same product common^(^ ^16^ ^)^. In 2012, an Australian and New Zealand survey found that less than half of packaged foods could be described as healthy using a nutrient profiling tool^(^ ^17^ ^)^.

In 2011–12, 63 % of Australian adults and 25 % of children were overweight or obese, and 35 % of the population’s total daily energy intake came from energy-dense nutrient-poor ‘discretionary foods’ that are high in added sugars, fats or salt^(^ ^18^ ^)^. These foods are more likely to be classified as UPF. Public health professionals agree that marketing of unhealthy foods, including via packaging, plays a role^(^ ^3^ ^,^ ^19^ ^–^ ^21^ ^)^.

The Australian and New Zealand food regulatory system aims to protect public health and safety by providing sufficient information, preventing misleading information and promoting healthy food choices^(^ ^22^ ^)^, while supporting an internationally competitive food industry^(^ ^23^ ^)^. Under the system, labels on packaging can display nutrition and health benefits; for example, using statements or claims permitted by the Australia New Zealand Food Standards Code (referred to as the ‘Food Code’ hereafter)^(^ ^24^ ^)^. The Australian Government’s voluntary front-of-package Health Star Rating labelling system (HSR) was launched in 2014 to assist consumers to select healthier foods^(^ ^25^ ^,^ ^26^ ^)^.

Regulating food marketing on product packaging, including the label, is a challenging food policy issue of public health significance^(^ ^27^ ^)^. Many food companies make corporate social responsibility commitments, particularly regarding safeguarding children from problems associated with food marketing^(^ ^28^ ^)^, and provide voluntary nutrition information on food labels in addition to the mandatory nutrition information panel^(^ ^29^ ^)^. It is important to understand the application of marketing statements as well as the nutrition and health claims made by manufacturers of high-market-share packaged foods and their potential impact on food choice. The aim of the present study was to objectively evaluate voluntary nutrition and health labelling, claims and marketing techniques on high-market-share UPF in Australia for their potential impact on public health.

## Methods

### Selection of food companies

The global network International Network for Food and Obesity Research Monitoring and Action (INFORMAS) aims to monitor, benchmark and support actions to create healthy food environments to reduce obesity, non-communicable diseases and their related inequalities^(^ ^30^ ^)^. It recommends focusing on the companies with the largest potential to impact public health nutrition when monitoring the policies and practices of the food industry^(^ ^31^ ^)^. Five high-market-share manufacturers of packaged foods in Australia were identified from Nielsen’s Top Brands Report 2009, specifically: Allen’s, Kellogg’s, Nestlé, Sanitarium and Uncle Toby’s^(^ ^32^ ^)^. Nestlé (including the Allen’s brand) had the largest share (13·9 %) of the chocolate and confectionery market in Australia^(^ ^33^ ^)^. Kellogg’s (17·8 %) had the largest market share of breakfast cereals in Australia, and Sanitarium (15·4 %) and Nestlé (including Uncle Toby’s; 7·1 %) also had a significant share^(^ ^34^ ^)^. To explore the emerging trend of supermarket own brands, the widely available Woolworths Supermarkets’ Macro range was also included^(^ ^35^ ^)^.

### Selection of packaged foods

Breakfast cereals, snacks and confectionery are among the categories most commonly marketed to children^(^ ^36^ ^,^ ^37^ ^)^. Foods audited included all the breakfast cereals, snacks and confectionery items, and selected beverages, condiments and liquid breakfast meal replacements (referred to as ‘meal replacements’ hereafter) available at the time of the study from the food manufacturers. Products were identified from the companies’ websites. Labelling information from the 230 packaged foods identified was collected.

### Data collection

The information for the audit was gathered from the companies’ or online shopping websites for Coles and Woolworths, and ‘in store’ at Coles and Woolworths supermarkets in Cockburn Gateway Shopping Centre in Western Australia, after obtaining permission from the store managers. The following information was collected: product name and brand, processed food group, added sugar and added fat ingredients, nutrition composition; the extent of packaging promotion to children; and nutrition labelling practices and price. Data collection was completed in September 2015.

### Categorisation of nutrition-related information

The extent of food processing for all packaged foods was identified, and foods were classified using the NOVA system^(^ ^12^ ^)^ to analyse the impact of these foods on public health- and diet-related outcomes. The NOVA system of classifying foods according to the extent of food processing, not nutrient content, aims to address the significance of industrial food processing to public health^(^ ^12^ ^)^. The term ultra-processed foods (UPF) is used to describe nutritionally poor, industrially processed foods that include cosmetic or sensory additives such as colours, flavours, sweeteners or processing aids; or undergo industrial processes which have no domestic equivalent such as extrusion^(^ ^12^ ^)^. The other groups in this classification system are: unprocessed or minimally processed foods, which may be consumed by themselves; processed culinary ingredients, which are used in food preparation; and processed foods, which are relatively simple foods with few ingredients^(^ ^12^ ^)^.

Free sugars and fats are commonly added to UPF^(^ ^12^ ^)^, and the Food Code definition of added sugars^(^ ^38^ ^)^ and a list of commonly used names for sugars^(^ ^39^ ^)^ were used to guide identification of added sugars.

**Fig. 1:**
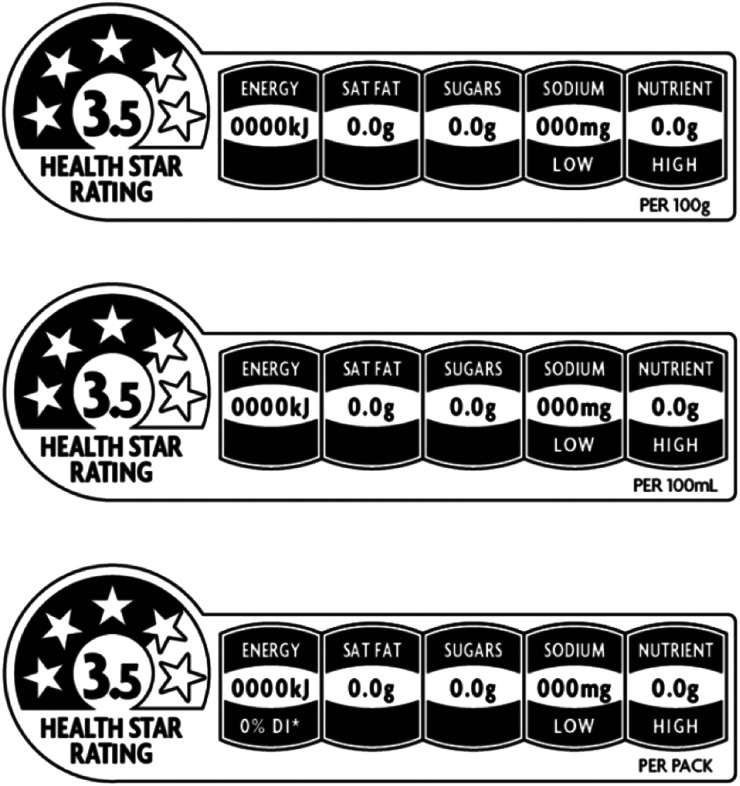
Health Star Rating front-of-pack device^(^ ^16^ ^)^

Guidelines for use of the voluntary HSR front-of-pack labelling device^(^ ^40^ ^)^ were used to assess the HSR on-pack. The HSR algorithm awards points for positive food or nutrient content (dietary fibre, protein and the proportion of fruit, vegetables, nuts and legumes) and subtracts points for negative nutrients (saturated fat, sodium, total sugars, but not added sugars), then assigns a score from 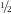 star to 5 stars, with 5 stars indicating the healthiest choice^(^ ^41^ ^)^ (Fig. 1). The online calculator provided on the HSR website^(^ ^40^ ^)^ was used to calculate the HSR for all products, using the nutrition information panel provided on the packaging. Few products included fruit, vegetables, nuts and legumes in the ingredients list; thus the calculation was based on content per 100 grams of the following: energy (kilojoules), saturated fat, sugars, sodium, dietary fibre and protein^(^ ^41^ ^)^.

### Classification of packaging information

**Fig. 2:**
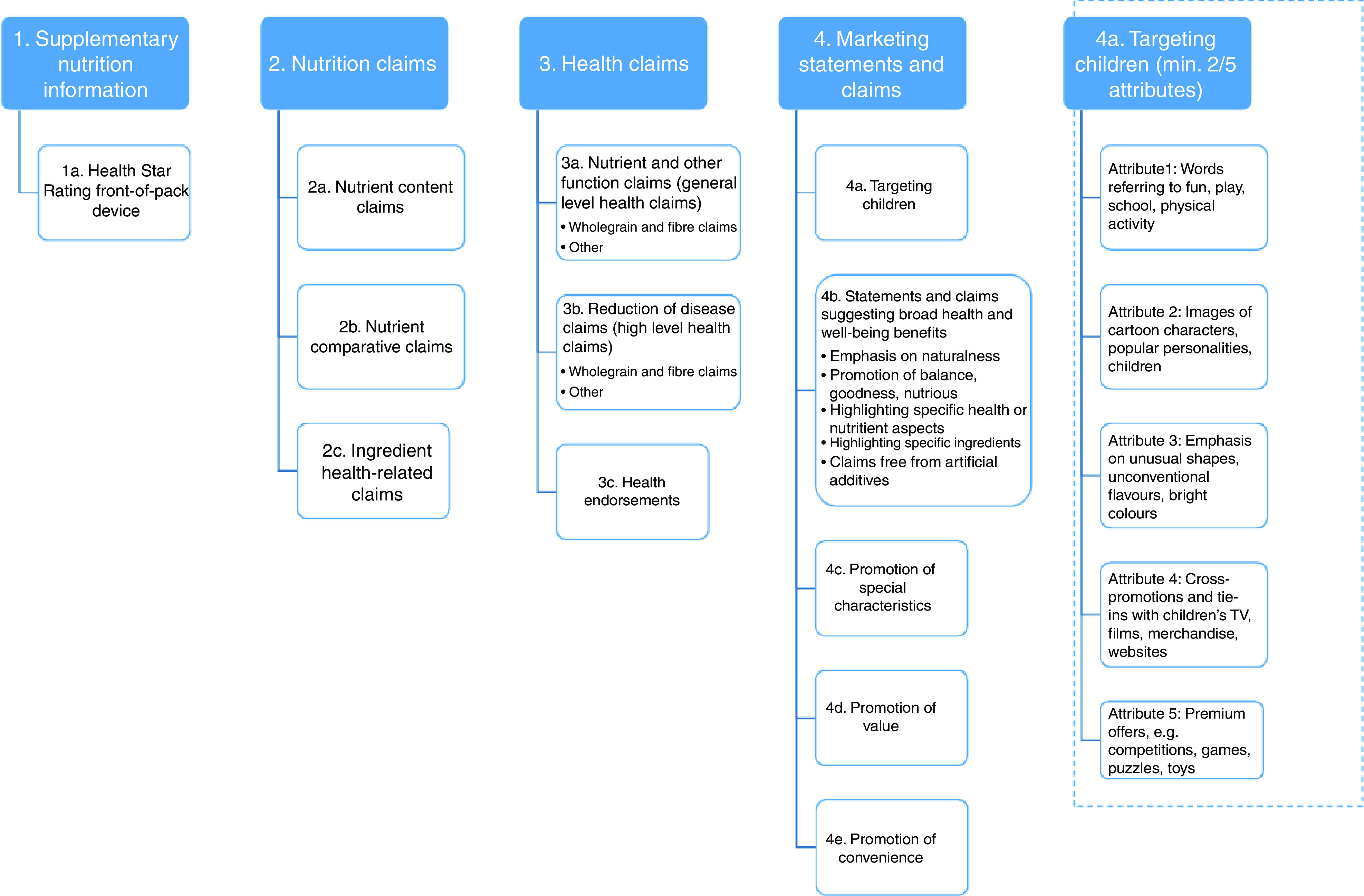
Taxonomy of nutrition- and health-related packaging information*. *Adapted from the INFORMAS food labelling taxonomy^(^ ^42^ ^)^, Mayhew *et al*.’s definitions of marketing techniques promoting health and well-being^(^ ^43^ ^)^, and Mehta *et al*.’s work defining food packaging targeting children^(^ ^3^ ^)^ (TV, television)

Packaging information was classified using the taxonomy shown in Fig. 2, based on defined nutrition information and marketing techniques identified by INFORMAS^(^ ^42^ ^)^ and Mayhew *et al*.^(^ ^43^ ^)^. The analysis identified the voluntary components implemented by the food companies that could be influenced by company corporate social responsibility policies. The presence of mandatory nutrition- and health-related information (e.g. nutrition information panels) was collected but not reported as they were present for all products. Products were classified as targeting children using criteria employed by Mehta *et al*. to examine packaging targeting Australian children, which stipulates a minimum of two out of a possible five attributes are present^(^ ^3^ ^)^ (Fig. 2).

### Compliance of statements and claims

The Food Code was used to assess legal compliance of food packaging information using the criteria that are required to be met to make health and nutrition claims. Claims were classified as: (i) nutrient content; (ii) nutrient comparative; (iii) ingredient health-related; (iv) general level health; or (v) high level health^(^ ^24^ ^,^ ^38^ ^,^ ^44^ ^)^. Health endorsements administered by organisations such as the Heart Foundation could not be assessed for accuracy, as criteria and product accreditation status were not publicly available. The Australian Competition and Consumer Commission’s food descriptors guideline to the Trade Practices Act 2006^(^ ^45^ ^)^, which defines deceptive and misleading representations about food and beverages, was referred to and mainly related to application of the Food Code for this data set.

Consideration was given to Clause 10 of the nutrition, health and related claims standard (Standard 1.2.7^(^ ^24^ ^)^), which states that it does not prescribe the words that must be used. Clause 13 of the nutrition, health and related claims standard (Standard 1.2.7^(^ ^24^ ^)^) states that nutrition content claims may be made about a property not listed in the Schedule (Schedule 4^(^ ^38^ ^)^) but the claim can only state that the food does or does not contain this property, that it contains a specified amount, or a combination of these two statements. Claims about the presence or amount of wholegrains were therefore determined to be permitted even though they were not specifically listed in Schedule 4, and were categorised as ingredient health-related claims for the present study. The Grains & Legumes Nutrition Council™ in Australia has created a voluntary code of practice to encourage promotion of wholegrains on food labels^(^ ^46^ ^)^. Therefore, the Grains & Legumes Nutrition Council’s criteria were used for assessment of claims against voluntary standards to assess compliance with industry self-regulation.

Data were analysed using the statistical software package IBM SPSS Statistics for Windows version 24 (released 2016).

## Results

### Level of food processing

Most (94 %) products were classified as UPF using the NOVA system^(^ ^12^ ^)^ (Table 1). These 215 UPF formed the data set for analysis.

**Table 1:** Packaging claims and statements present on ultra-processed foods suitable for families from four Australian manufacturers, September 2015

	Breakfast cereals			Beverages			Condiments			Confectionery			Snacks			Meal replacements			All products		
	*n*	%	Mean	*n*	%	Mean	*n*	%	Mean	*n*	%	Mean	*n*	%	Mean	*n*	%	Mean	*n*	%	Mean
NOVA classification of food processing																					
Group 1 (unprocessed)	8	8·70	–	0	0·00	–	0	0·00	–	0	0·00	–	1	1·72	–	0	0·00	–	9	3·91	–
Group 2 (processed culinary ingredients)	0	0·00	–	0	0·00	–	2	28·57	–	0	0·00	–	0	0·00	–	0	0·00	–	2	0·87	–
Group 3 (processed foods)	2	2·17	–	0	0·00	–	0	0·00	–	0	0·00	–	2	3·45	–	0	0·00	–	4	1·74	–
Group 4 (ultra-processed foods)	82	89·13	–	15	100·00	–	5	71·43	–	46	100·00	–	55	94·83	–	12	100·00	–	215	93·48	–
Proportion of final data set	82	38·14	–	15	6·98	–	5	2·33	–	46	21·40	–	55	25·58	–	12	5·58	–	215	100·00	–
Products containing added sugar																					
No. of products	78	95·12	–	12	80·00	–	3	60·00	–	46	100·00	–	53	96·36	–	12	100·00	–	201	94·88	–
Mean sugar content (g)	–	–	19·5	–	–	29·6	–	–	6·8	–	–	51·5	–	–	24·8	–	–	7·1	–	–	27·4
Mean no. of terms used for added sugar	–	–	2·6	–	–	0·9	–	–	0·6	–	–	1·8	–	–	3·8	–	–	2·3	–	–	2·5
Products containing added fat																					
No. of products	31	37·80	–	5	33·33	–	4	80·00	–	30	65·22	–	52	98·11	–	12	100·00	–	134	62·33	–
Mean fat content (g)	–	–	4·9	–	–	2·8	–	–	42·6	–	–	14·1	–	–	9·7	–	–	1·4	–	–	8·6
Mean no. of terms used for added fat	–	–	0·4	–	–	0·7	–	–	0·8	–	–	1·1	–	–	1·6	–	–	2·0	–	–	1·0
Australian Guide to Healthy Eating indicator																					
kJ per serving is≥600	39	47·56	–	2	13·33	–	0	0·00	–	2	4·35	–	7	12·73	–	12	100·00	–	62	28·84	–
1. Supplementary nutrition information present																					
1a. Health Star Rating device present	82	100·00	–	10	66·67	–	5	100·00	–	0	0·00	–	9	16·36	–	12	100·00	–	118	54·88	
Mean calculated Health Star Rating	–	–	3·9	–	–	3·2	–	–	3·6	–	–	1·2	–	–	2·6	–	–	4·6	–	–	3·0
2. Nutrition claims	70	85·37	–	11	73·33	–	5	100·00	–	4	8·70	–	19	34·55	–	12	100·00	–	121	56·28	–
2a. Nutrient content claims	66	80·49	–	11	73·33	–	5	100·00	–	2	4·35	–	16	29·09	–	12	100·00	–	112	52·09	–
2b. Comparative nutrient claims	5	6·10	–	5	33·33	–	0	0·00	–	2	4·35	–	3	5·45	–	7	58·33	–	22	10·23	–
2c. Ingredient health-related claims	47	57·32	–	0	0·00	–	0	0·00	–	0	0·00	–	4	7·27	–	0	0·00	–	51	23·72	–
3. Health claims	44	53·66	–	1	6·67	–	2	40·00	–	0	0·00	–	4	7·27	–	2	16·67	–	53	24·65	–
3a. General level health claims	16	19·51	–	1	6·67	–	0	0·00	–	0	0·00	–	0	0·00	–	2	16·67	–	19	8·84	–
3b. High level health claims	1	1·22	–	0	0·00	–	2	40·00	–	0	0·00	–	0	0·00	–	0	0·00	–	3	1·40	–
3c. Endorsements	37	45·12	–	0	0·00	–	2	40·00	–	0	0·00	–	4	7·27	–	0	0·00	–	43	20·00	–
4. Marketing statements or claims	79	96·34	–	14	93·33	–	5	100·00	–	44	95·65	–	55	100·00	–	12	100·00	–	209	97·21	–
Mean no. of marketing attributes present (4a–4e)	–	–	2·4	–	–	2·1	–	–	3·0	–	–	2·8	–	–	3·1	–	–	2·3	–	–	2·6
4a. Packaging designed to appeal to children	25	30·49	–	4	26·67	–	4	80·00	–	28	60·87	–	40	72·73	–	0	0·00	–	101	46·98	–
Mean no. of children’s marketing attributes present (max. 5)	–	–	1·5	–	–	1·3	–	–	1·8	–	–	2·0	–	–	2·2	–	–	1·0	–	–	1·8
4b. Statements suggesting broad health benefits	75	91·46	–	13	86·67	–	5	100·00	–	44	95·65	–	49	89·09	–	12	100·00	–	198	92·09	–
Emphasis on naturalness	19	23·17	–	0	0·00	–	0	0·00	–	7	15·22	–	7	12·73	–	0	0·00	–	33	15·35	–
Promotion of balance, goodness, nutritious	43	52·44	–	5	33·33	–	0	0·00	–	42	91·30	–	25	45·45	–	7	58·33	–	122	56·74	–
Highlighting specific health or nutrient aspects	29	35·37	–	8	53·33	–	3	60·00	–	0	0·00	–	15	27·27	–	12	100·00	–	67	31·16	–
Highlighting specific nutrients	30	36·59	–	1	6·67	–	4	80·00	–	0	0·00	–	31	56·36	–	2	16·67	–	68	31·63	–
Claims free from artificial additives	26	31·71	–	4	26·67	–	4	80·00	–	32	69·57	–	34	61·82	–	0	0·00	–	100	46·51	–
4c. Promotion of special characteristics	5	6·10	–	9	60·00	–	0	0·00	–	19	41·30	–	17	30·91	–	0	0·00	–	50	23·26	–
4d. Promotion of value	1	1·22	–	0	0·00	–	0	0·00	–	0	0·00	–	0	0·00	–	0	0·00	–	1	0·47	–
4e. Promotion of convenience	15	18·29	–	0	0·00	–	0	0·00	–	1	2·17	–	0	0·00	–	7	58·33	–	23	10·70	–

### Added sugars and added fats

Most UPF products (95 %) contained added sugars (Table 1). Fourteen types of sugar were used in the products, with thirty-four different ingredient names used (e.g. ‘sugar’ was also listed as ‘raw sugar’, ‘organic raw sugar’, ‘organic sugar’, ‘cane sugar’ and ‘brown sugar’). The overall mean number of ingredient names used for sugar per product was 2·5 (range 0–8). The mean number of ingredient names used for sugar per pack was highest for snack foods at 3·8 (range 0–8). Over half (62 %) of products contained added fats; however, the mean number of terms used in ingredients lists was only 1·0 (range 0–3; Table 1).

### Classification of packaging information

The numbers of products providing supplementary nutrition information (i.e. HSR), nutrition claims, health claims, and marketing statements or claims are shown in Table 1. Overall 55 % of products had a HSR, 59 % had nutrition or health claims, and 97 % had selected marketing techniques. On average, each product displayed 1·5 (range 0–10) health or nutrition claims and 2·6 (range 0–5) marketing techniques on the packaging.

The mean number of health stars for all products was 2·97 HSR (range 0·5–5). Breakfast cereals, condiments and meal replacements had a mean HSR of 3·5–5·0 (Table 1) and 55 % of all products achieved a HSR of 3·5–5·0.

Most (95 %) of the products with health or nutrition claims also included marketing statements highlighting broad health benefits. Of these, 87 % also featured the HSR; and 82 % would be described as healthy (HSR of 3·5–5·0) based on research that determined that foods with these HSR scores were more likely to be consistent with the nutritious core foods recommended by the Australian Guide to Healthy Eating (AGHE)^(^ ^47^ ^–^ ^49^ ^)^.

The most frequent marketing technique used was promotion of ‘balance’ or ‘goodness’ (57 %), followed by claims of being free from artificial additives (47 %) and packaging that targets children (47 %). Promotion of value or convenience were the least used marketing techniques.

Most (61 %) of the packaging targeting children featured three of the five identified marketing attributes. Fewer products designed to appeal to children featured the HSR (35 %) compared with family-oriented products (55 %).

### Validation of statements and claims

Results from validation of the HSR and nutrition and health claims are summarised in Table 2. The HSR device was used on 55 % of products and the calculation was correct for all products.

**Table 2:** Accuracy of packaging information present on ultra-processed foods suitable for families from four Australian manufacturers, September 2015

	No. of products making the claim*	No. of products with all claims correct	% of products with all claims correct
1. Supplementary nutrition information			
1a. Health Star Rating device	118	118	100·0
2. Nutrition claims present	118	21	17·8
2a. Nutrient content claims	113	21	18·6
Contains/source/good source of fibre	44	5	11·4
High/very high/excellent source of fibre	24	3	12·5
Other nutrient content claim†	95	21	22·1
2b. Comparative nutrient claims	22	6	27·3
2c. Ingredient health-related claims	52	38	73·1
Contains/source/amount of wholegrains	40	38	95·0
High/good source of wholegrains	7	0	0·0
Very high/excellent source of wholegrains	5	0	0·0
Contribution to daily wholegrains target	10	8	80·0
3. Health claims present	19	15	78·9
3a. General level health claims	17	13	76·5
General level claim: fibre or wholegrain	3	3	100·0
General level claim: other	16	12	75·0
3b. High level health claims	3	3	100·0
High level claim: fibre or wholegrain	1	1	100·0
High level claim: other	2	2	100·0
3c. Endorsements	43	n/a	n/a

n/a, not applicable.

* Each item of packaging can include multiple classifications of nutrition and health claims.

† An additional 16·3 % of claims could not be assessed for accuracy as micronutrient content data were not collected.

Nutrition claims were correct for 18 % of products making this type of claim (Table 2). Few claims about fibre content complied with criteria specified in the Food Code. The minimum quantity specified in the claims criteria was often not met. Claims about presence of wholegrains were appropriately specified by 73 % of products, and substantiated with the wholegrain ingredients identified in ingredients lists. However, the criteria stipulated by the Grains & Legumes Nutrition Council industry group for high or very high source of wholegrains claims were not met by any of the products making these claims.

Other nutrient claims were common (44 %) with some relating to micronutrient content; 22 % of these claims met Food Code criteria (Table 2). Unspecific wording was the most common issue, such as ‘contains B vitamins’ without giving details of the individual B vitamins.

Only 27 % of comparative nutrient claims met Food Code criteria (Table 2). Again, wording was not specific enough; for example, ‘40 % less sugar when compared to leading kids snacks’ without specifying the products being compared.

Health claims were present on 25 % of products and were correct for 79 % (Table 2). The most frequent health statement was through a third-party endorsement logo such as the Heart Foundation Tick (20 %). There were much higher levels of compliance for general level and high level claims than for nutrition claims, with most meeting criteria (77 % of general level, 100 % of high level). Two general level health claims were unable to be assessed as they referred to health benefits for nutrients that were not included on packaging as part of the nutrition information panel.

## Discussion

The current study identified packaging information present on UPF promoting nutrition and health, and classified it using a taxonomy based on previous work in this area^(^ ^3^ ^,^ ^42^ ^,^ ^43^ ^)^. The presence of added sugars and fats, and ingredient labelling practices were investigated. Use of the HSR, nutrition and health claims, and marketing techniques was also investigated. Prevalence of nutrition and health attributes on packaging specifically targeting children was of particular interest.

### Use of the taxonomy for classifying nutrition and health statements and claims

The taxonomy of nutrition and health statements and claims adapted for use in the present study provided a framework for classifying the information present on high-market-share UPF in Australia. The novel aspect of our study is the integration of a food labelling taxonomy from INFORMAS^(^ ^42^ ^)^, marketing techniques promoting health and well-being^(^ ^43^ ^)^, and food packaging targeting children^(^ ^3^ ^)^ to describe the nature and extent of this information.

### Added sugars and fats

The present study identified a high prevalence of added sugars in UPF. This is not surprising, as Australian and US population dietary surveys^(^ ^50^ ^,^ ^51^ ^)^ have found UPF contribute most of the added sugars consumed. An independent review of Australian food labelling recommended that changes are made to the way added fats and added sugars are identified in ingredients lists, to improve transparency:

‘Where sugars, fats or vegetable oils are added as separate ingredients in a food, the terms “added sugars” and “added fats” and/or “added vegetable oils” be used in the ingredient list as the generic term, followed by a bracketed list (e.g., added sugars (fructose, glucose syrup, honey), added fats (palm oil, milk fat) or added vegetable oils (sunflower oil, palm oil)).’ (Recommendation 12, p. 9^(^ ^52^ ^)^)

Multiple terms for added sugars were commonly used on packaging, which makes deciphering ingredients lists difficult for consumers. Splitting sugar into component ingredients places them lower in the list of ingredients, obscuring the ranking that sugar would otherwise have. Our findings support the recommendation for increased transparency of added sugars on packaging^(^ ^52^ ^)^. A separate added sugars line on nutrition information panels, as has recently been introduced in the USA^(^ ^53^ ^)^, should also be considered. Interestingly, despite the recommendation for similar action on added fats^(^ ^52^ ^)^, the present study found that they were more clearly labelled.

The majority of Australian adults and children consume too much added sugar, typically consumed as UPF^(^ ^54^ ^)^. Governments^(^ ^55^ ^)^, public health researchers^(^ ^56^ ^)^, campaigners^(^ ^57^ ^)^ and even supermarket chains^(^ ^58^ ^)^ have called for measures to control or reduce the amount of added sugars present in processed foods. Clearly identifying the amount of added sugars present in UPF is a priority to assist food regulation to protect public health by informing consumers and to underpin health promotion interventions. For example, the LiveLighter^©^ social marketing campaign aims to educate the population about the amount of sugar present in soft drinks^(^ ^59^ ^)^ and public health advocates are calling for a sugar tax on soft drinks in Australia^(^ ^60^ ^)^.

### Classification of packaging information

UPF have been described as hyper-palatable products that are attractively packaged and aggressively marketed, including making use of health statements and claims^(^ ^12^ ^)^. The present study has demonstrated the accuracy of the definition when applied to a sample of high-market-share UPF in Australia. More than half of the UPF packaging in our study featured nutrition or health claims, and almost all of the packaging utilised marketing techniques which related to nutrition and health. In addition, each pack typically displayed multiple claims and marketing techniques, demonstrating the extent to which this sort of information is used. Analysis from Canada, the UK, the USA and Brazil has demonstrated the poor nutritional quality of UPF^(^ ^61^ ^–^ ^63^ ^)^, so this high prevalence of nutrition- and health-related statements and claims on packaging is concerning.

Over half of the products selected for the current study featured a HSR on the packaging, although monitoring surveys at the time reported that only 3 % of products carried a HSR^(^ ^64^ ^)^. Breakfast cereal manufacturers adopted the HSR faster than other categories^(^ ^64^ ^)^, which is not surprising given that Sanitarium, a breakfast cereal manufacturer, and Woolworths Supermarkets were the first public commercial supporters of the scheme^(^ ^64^ ^,^ ^65^ ^)^.

The present study demonstrates the complexity of attempting to consolidate different principles for defining and identifying healthy food choices, for example based on nutrient profiling (HSR), food group categorisation (AGHE) or processing (NOVA). The HSR of most UPF products that featured nutrition or health claims in the current study was 3·5–5·0; previous research suggested that foods rated 3·5 stars or above were consistent with the nutritious core foods in the AGHE food selection guide^(^ ^47^ ^–^ ^49^ ^)^. The level of HSR, and the presence of nutrition and health claims, on UPF is at odds with their typically poor nutritional quality^(^ ^61^ ^)^. Another Australian study found similar anomalies; for example, bread or pasta, classified as UPF due to their level of processing, are considered nutritious core foods in the AGHE^(^ ^66^ ^)^.

A recent review of the relationship between changes in the food system and the global nutrition transition highlights the challenges and importance of describing and categorising foods to measure the health implications of the ongoing changes in the food supply^(^ ^67^ ^)^. In 2015 Poti *et al.* extended the NOVA system by further describing food processing and including ‘convenience’, dividing UPF into two groups: ‘highly processed’ ingredients and ‘highly processed’ stand-alone foods^(^ ^13^ ^)^. Resolution of discrepancies in recommended dietary patterns such as those of the AGHE and individual foods recommended by food processing systems such as NOVA, as well as front-of-pack labelling advice including HSR and nutrition and health claims, is needed to clarify dietary advice to consumers. Further research to develop an understanding of the effect of multiple nutrition and health claims and statements, combined with the HSR, on consumer food selection is also suggested.

Most products that featured nutrition or health claims also carried messages that were classified as marketing techniques. Marketing techniques designed to make products appealing to potential consumers do not receive the same level of regulatory scrutiny as claims. A wide range of marketing techniques was evident in the present study, with most statements suggesting broad health benefits. These marketing techniques were applied to packaging in all categories surveyed, including confectionery and snacks. This is consistent with recent research conducted across sixteen countries which found that 87 % of all snack food packaging featured claims emphasising general health, well-being or naturalness^(^ ^43^ ^)^. Unregulated statements that products are ‘free from’ artificial additives such as colours and flavours, or promote ‘balance’ or ‘goodness’, often mislead consumers into thinking these products are more healthful than they actually are^(^ ^43^ ^)^ or that their inclusion in a healthy diet is permitted or normal^(^ ^9^ ^)^. These are common marketing techniques used by UPF manufacturers to broaden their appeal and make frequent consumption acceptable^(^ ^12^ ^)^. Our findings suggest that urgent action is needed to prevent marketing practices that potentially mislead consumers into thinking these unhealthy products are healthy.

The current study adds to the existing literature documenting the high level of inappropriate marketing to children present on packaging of UPF in Australia^(^ ^3^ ^)^. Most products that were designed to appeal to children featured three of the five marketing attributes previously identified^(^ ^3^ ^)^. Voluntary action by the food industry to restrict marketing of food to children was initiated by the Australian Food and Grocery Council in 2008^(^ ^68^ ^)^. However, the Responsible Children’s Marketing Initiative^(^ ^68^ ^)^ focuses on encouraging responsible advertising and to date has not addressed marketing at the point of sale, including packaging. The voluntary approach has also not yet proved to be effective in reformulating products targeting children to improve their nutritional quality^(^ ^69^ ^,^ ^70^ ^)^. Most parents express concern about the level of food marketing to children^(^ ^71^ ^)^. Therefore, more public policies are needed to assist parents to identify healthy packaged foods. These policies should address the accuracy and quality of nutritional information provided on UPF.

Given the prevalence of marketing techniques identified in our study, and the challenges in regulating packaging on products targeting children^(^ ^27^ ^)^, alternative strategies to assist consumers to select healthy packaged foods could be investigated. For example, the supermarket-wide Guiding Stars system uses an algorithm to assess both positive and negative nutrient content and has been adopted by five supermarket chains in the USA^(^ ^72^ ^)^. Guiding Stars aimed to overcome consumers’ inability to make sense of the plethora of information present on food packaging by providing a simple guide on the shelf-edge tag along with the price^(^ ^73^ ^)^. Evaluation shows the Guiding Stars shelf-edge labelling of healthy foods was effective in assisting consumers to purchase more healthy foods overall^(^ ^74^ ^)^. Australian public policy to assist consumers to select healthy packaged foods should consider such strategies that can be applied across all UPF available in supermarkets, particularly if voluntary uptake of HSR does not prove effective in assisting consumers to select healthy foods.

### Validation of statements and claims

The present study was unique in that it validated the HSR and nutrition and health claims present on UPF against the Food Code^(^ ^24^ ^,^ ^29^ ^,^ ^38^ ^,^ ^44^ ^)^ and other criteria^(^ ^41^ ^,^ ^46^ ^)^. Findings showed that the HSR and high level health claims used were typically accurate. However, there were many issues identified for nutrition claims, and lower levels of accuracy for general level health claims.

Claims on breakfast cereals about dietary fibre or wholegrains content were present on some packaging; however, many were not accurate because the minimum quantity specified in the claims criteria in the Food Code was not met^(^ ^38^ ^)^. This finding is surprising, as the packaging included in the current study was from high-market-share food manufacturers who would be expected to meet the criteria specified in the Food Code. In addition, claims about products being a high or very high source of wholegrains not only failed to follow the Food Code^(^ ^29^ ^)^, but also failed to adhere to the industry’s voluntary code^(^ ^46^ ^)^. This indicates the importance of monitoring and surveillance of packaging information applied to UPF, with financial penalties for lack of adherence to regulations and guidelines.

UPF failing to provide accurate nutrition claims on packaging included wording that was not specific enough, typically when products declared the presence of added vitamins and minerals, or made comparisons of nutrient content with other products. However, for the information to be helpful to consumers it needed to include details that were not provided. These deceptive and misleading practices should be addressed in public policies to provide consumer-friendly nutrition labelling that is easy to understand and addresses public health concerns^(^ ^75^ ^)^.

### Limitations

The present study has a number of strengths and limitations. Challenges were faced in determining the accuracy of claims for various reasons. Clause 10 of the nutrition, health and related claims standard (Standard 1.2.7^(^ ^24^ ^)^) states that it does not prescribe the words that must be used. Therefore, assessment of the accuracy of these statements made on packaging was open to interpretation, and other researchers or enforcement authorities may differ in their views.

The study’s findings are likely generalisable to breakfast cereals, snacks and confectionery in the Australian food supply, and given the globalised supply of multinational UPF, may be applicable to other countries^(^ ^10^ ^)^. Only 215 UPF products in five food categories were audited; however, it is likely that the same issues apply across other food categories or with other food manufacturers. Therefore, we recommend further research to classify packaging information from a broader range of product categories. Testing the accuracy of nutrition and health claims on a larger sample of products would also assist in identifying the scale of the problems identified in the current study. Packaging information including the food industry’s Daily Intake Guide thumbnail^(^ ^76^ ^)^ and micronutrients present in nutrition information panels were not collected in the present study. Future research should include this information so that full assessment of supplementary nutrition information (i.e. HSR for the present study) and nutrition and health claims can be undertaken.

Strengths of the study include the detailed taxonomy applied to classify packaging information, which includes nutrition and health claims, marketing techniques and classification of products designed to appeal to children, as well as validating these nutrition and health statements and claims.

## Conclusions

The taxonomy of nutrition and health statements and claims proved effective in describing the nature and extent of information present on packaging of high-market-share UPF in Australia. Based on the findings in the present study, UPF were typically attractively packaged with labels that incorporated multiple marketing techniques, and extensively utilised nutrition and health statements and claims, despite many products containing added sugars or being rated a less healthy choice. The proportion of inappropriate or inaccurate statements and claims is concerning, particularly on UPF packaging designed to appeal to children. Public policies to assist parents to select healthy packaged foods need to address the accuracy and quality of nutritional information provided on packaged foods, reducing deceptive marketing practices. Recommendations include: clearly identifying the amount of added sugars present in UPF by adding a separate added sugars line on nutrition information panels similar to the USA; conducting further research to ensure the HSR correctly identifies the nutritional quality of UPF; conducting further research to build the evidence for the role of level of food processing in the selection of healthy dietary patterns; resolving discrepancies in recommended dietary patterns (e.g. AGHE) and individual foods recommended by different systems such as NOVA and front-of-pack labelling advice (e.g. HSR); and consider wider application of a modified HSR across all food products to more accurately advise consumers on how to select foods for a healthy dietary pattern. Monitoring and surveillance of compliance of packaging information applied to UPF with current regulations is also important.
